# Proinflammatory Epstein–Barr Virus Antibody Functions Track with Disease Activity in Multiple Sclerosis

**DOI:** 10.1002/ana.78285

**Published:** 2026-06-17

**Authors:** Malina Behrens, Paula Terroba‐Navajas, Eline A.J. Willemse, Sabine Schädelin, Manuel Comabella, Christian Münz, Gordan Lauc, Yannic Bartsch, Jens Kuhle, Jan D. Lünemann

**Affiliations:** ^1^ Department of Neurology University Hospital Münster Münster Germany; ^2^ Department of Neurology University Hospital and University of Basel Basel Switzerland; ^3^ Multiple Sclerosis Centre and Research Center for Clinical Neuroimmunology and Neuroscience (RC2NB), Department of Biomedicine and Clinical Research University Hospital and University of Basel Basel Switzerland; ^4^ Department of Clinical Research University Hospital Basel, University of Basel Basel Switzerland; ^5^ Servei de Neurologia, Centre d'Esclerosi Múltiple de Catalunya (Cemcat) Institut de Recerca Vall d'Hebron (VHIR), Hospital Universitari Vall d'Hebron, Universitat Autònoma de Barcelona Barcelona Spain; ^6^ Center for Networked Biomedical Research on Neurodegenerative Diseases (CIBERNED)–ISCIII CIBERNED Madrid Spain; ^7^ Viral Immunobiology, Institute of Experimental Immunology University of Zürich Zurich Switzerland; ^8^ Faculty of Pharmacy and Biochemistry University of Zagreb Zagreb Croatia; ^9^ Genos Ltd Glycoscience Research Laboratory Zagreb Croatia; ^10^ Laboratory of Anti‐Viral Antibody‐Omics, TWINCORE—Institute for Experimental Infection Research Helmholtz Center for Infection Research (HZI) and Medical School Hannover (MHH) Hannover Germany; ^11^ Division of Neurology, Department of Medicine University of British Columbia Vancouver BC Canada; ^12^ Djavad Mowafaghian Centre for Brain Health University of British Columbia Vancouver BC Canada; ^13^ School of Biomedical Engineering University of British Columbia Vancouver BC Canada

## Abstract

**Objective:**

Epstein–Barr virus (EBV) has been implicated in the pathogenesis of multiple sclerosis (MS). Elevated immunoglobulin G (IgG) titers against EBV nuclear antigen 1 (EBNA1) represent the most consistent serological marker of MS risk, with levels remaining persistently elevated following disease onset.

**Methods:**

We conducted high‐dimensional profiling of virus‐specific antibody‐associated immune features in 60 individuals with relapsing‐onset MS followed over time and in 54 age‐ and sex‐matched EBV‐seropositive healthy controls.

**Results:**

EBNA1‐specific IgG in MS patients exhibited a distinct proinflammatory Fc signature, characterized by increased affinity for Fc γ receptors (FcγRs) and enhanced Fc‐mediated effector functions, including antibody‐dependent phagocytosis, complement activation, and natural killer cell engagement. This signature was not observed for antibodies against other common viral antigens and showed a strong association with MS. Increased FcγR binding by IgG, specific for both EBNA1 and the lytic EBV glycoprotein 350 (EBV‐gp350) correlated with disease activity, defined as clinical relapse and/or presence of contrast‐enhancing brain lesions (CELs).

**Interpretation:**

Both fragment antigen‐binding and pro‐inflammatory functional Fc‐mediated features contribute to the association between EBNA1‐specific antibody responses and MS. Fc effector functions of EBV‐specific antibodies may actively participate in promoting focal disease activity in MS. ANN NEUROL 2026;100:547–558

Epidemiological, serological, and immunological studies in large, clinically well‐defined cohorts have consistently demonstrated a strong association between Epstein–Barr virus (EBV) infection and the risk to develop multiple sclerosis (MS).^1^, ^2^, ^3^, ^4^ Primary EBV infection, indicated by the emergence of EBV‐specific antibodies in previously seronegative individuals, but not infection with any other virus, significantly increases the risk of developing MS.^2^ Among serologic markers, elevated immunoglobulin G (IgG) titers against EBV nuclear antigen 1 (EBNA1) are the most robust and consistent serologic risk factor for MS.^3^, ^4^, ^5^ After disease onset, high EBNA1‐specific IgG levels remain a prominent immunological feature of MS.^6^, ^7^, ^8^, ^9^, ^10^

The mechanisms by which EBNA1‐specific IgG may contribute to MS susceptibility are incompletely understood, but might include cross‐reactivity with MS‐associated antigens expressed in the central nervous system (CNS).^11^, ^12^, ^13^, ^14^, ^15^, ^16^ Moreover, whether EBNA1‐ and EBV‐specific antibody responses are associated with MS activity after disease onset remains poorly characterized.

Antibodies contribute to the control of viral infections via interacting with the innate and adaptive immune system, supporting sequestration and uptake of pathogens, increased antigen presentation, and regulation of inflammation. The diverse effector functions that are deployed by antibodies are mediated by both the antigen‐binding F(ab)_2_ and the constant Fc domain, which provides specific instructions to the immune system through ligation of Fc receptors or binding of the complement component C1q.^17^

The goal of the present study was systematically to profile the biochemical and functional landscape of EBNA1‐specific antibodies in patients diagnosed with MS using an unbiased, high‐dimensional serology platform and to investigate whether EBV‐IgG biosignatures are associated with MS disease activity.

## Methods

### Study Participants

The “focal inflammation” subcohort from the Swiss Multiple Sclerosis Cohort (SMSC; NCT02433028) was used in this study.^18^ This prospective, multicenter cohort study was conducted across 8 Swiss academic medical centers.^19^ It was carried out between January 1, 2012 and October 22, 2022 and approved by the ethics committees of all participating centers. Written informed consent was obtained from all participants before inclusion. This study followed the Strengthening the Reporting of Observational Studies in Epidemiology (STROBE) reporting guidelines. Clinical, demographic and neuroimaging data as well as blood samples were collected at regular intervals (every 6 or 12 months) following a standardized protocol. Certified raters performed clinical assessments, including Expanded Disability Status Scale (EDSS) scoring.^19^, ^20^ The samples were acquired from each individual during both active disease phase (MS‐Active, defined as clinical relapse and/or contrast‐enhancing brain lesions [CELs]) and remission (MS‐Remission) phase. The temporal order of sampling was not uniform across patients. In some individuals, remission preceded active disease sampling, whereas in others active disease was sampled first. This distribution minimizes potential bias related to a fixed sampling sequence.

Patients classified as being in an active disease phase had experienced a clinical relapse within the prior 30 days and/or exhibited 1 or more CELs on magnetic resonance imaging (MRI) less than 30 days before serum sampling. For remission time points, samples were excluded if obtained within 1 year before or 6 months after a relapse or if a CEL had been detected on an MRI within 30 days of sampling. All time points were independently reviewed by 2 neurologists to confirm objective disease activity and exclude cases with clinical worsening not adequately reflected by EDSS (eg, objectively worsening upper limb ataxia despite stable EDSS scores). Patients with relevant comorbidities (eg, diabetes mellitus, hypertension, or orthopedic surgery impacting walking distance) were excluded. Based on these criteria, patients with the most pronounced signs of active disease or clearly defined remission were selected from the SMSC at the University Hospital Basel. When multiple eligible samples were available, prioritization followed a predefined hierarchy: samples with the highest number of gadolinium (Gd)+ lesions were selected, and if equal, samples with a more recent relapse were preferred, and if still multiple candidates remained, the sample obtained closest to the MRI was chosen to favor temporal proximity. Age matching within 0.3 standard deviations was implemented as an additional selection criterion. Potential samples obtained during corticosteroid treatment were excluded. Relapses were either treated with corticosteroids that had been stopped at least 3 months before biosampling or were initiated on the same day (after sampling; n = 2) or on subsequent days. For Gd+ lesions, no individually documented corticosteroid treatment overlapped with the interval between MRI acquisition and biosampling. No plasma exchange was performed before sampling. Twenty‐seven of 60 patients changed their treatment status during the follow‐up period. This was explicitly taken into account in the additional analyses, in which comparisons were limited to patients with stable treatment at both time points.

All participants included in the study were of White race and ethnicity. Other racial and ethnic subgroups were too small for meaningful analysis. Detailed information on patient selection and clinical assessments is provided in Table 1. Blood samples from healthy controls (HCs) with the same age and sex distribution were collected at the University Hospital Basel and at the University Hospital Münster, Germany. To minimize possible plate effects, samples from MS patients and HCs were distributed such that the same number of samples from each group was measured on each plate. In addition, paired samples (eg, from the same patient during active disease and remission) were always measured on the same plate to enable direct comparison.

**TABLE 1:** Patient Characteristics

Variable	MS‐Active	MS‐Remission	HC	*p*
No. of samples	60	60	54	NA
Sex				0.92
F	44 (73.3)		38 (70.4)	
M	16 (26.7)		16 (29.6)	
Age, median (IQR), yr	41.6 (33.3–47.2)	41.9 (32.8–47.9)	46.8 (30.3–56.2)	0.48
Disease category at study entry				>0.99
RRMS	56 (93.3)	56 (93.3)		
Progressive MS	4 (6.7)	4 (6.7)		
EDSS score, median (IQR)	2.0 (2.0–3.0)	2.0 (1.5–3.0)		0.30
Disease duration, median (IQR), yr	8.0 (3.8–17.0)	7.8 (3.9–16.0)		0.54
DMT				<0.01
Untreated	22 (36.7)	8 (13.3)		
Platform	7 (11.7)	5 (8.3)		
Oral	28 (46.7)	35 (58.3)		
Monoclonal antibody therapies	3 (5)	12 (20)		
Relapse within 30 days	31 (51.7)	0		NA
Relapse or lesion category				NA
Gd lesion	29 (48.2)	0		
Relapse	17 (28.3)	0		
Relapse and Gd lesion	9 (15.0)	0		
Relapse, no MRI	5 (8.3)	0		
Days since last relapse, median (IQR)	16 (4.5–22.0)	0		NA
T2w lesion volume, median (IQR), ml^a^	8.0 (2.6–19.6)	6.7 (2.5–15.0)		0.54
New/enlarging T2w lesion at sample^b^	4.0 (1.0–13.0)	1.0 (0.0–4.0)		0.006

From each patient, 2 samples were collected at different time points: 1 during remission (MS‐Remission) and 1 during active disease phases (MS‐Active).

CI =confidence interval; DMT =disease‐modifying treatment; EDSS = Expanded Disability Status Scale; F = female; Gd lesion = gadolinium‐enhancing lesion; IQR = interquartile range; M = male; MS = multiple sclerosis; RRMS = relapsing remitting MS; T2w lesion = T2‐weighted lesion; yr = year.

^a^ Data was available for 42 relapse and 47 remission patients.

^b^ Data was available for 25 relapse and 33 remission patients.

### Antibody Titer Analysis

EBNA1‐specific IgG antibody titers were quantified using the EBNA1 IgG enzyme‐linked immunosorbent assay (ELISA) kit (RE58741, Tecan IBL International, Hamburg, Germany) according to the manufacturer's instructions. Similarly, antibodies against the EBV capsid antigen (EBV‐CA) were measured using the EBV‐CA IgG ELISA kit (El 2,791‐9601G, Euroimmun, Lübeck, Germany) according to the manufacturer's recommendations.

### Biotinylation and Coupling of Beads

Recombinant EBNA1 (ab138345, abcam, Cambridge, UK) and influenza hemagglutinin (Influenza‐HA) proteins (IT‐003‐B11p; IT‐003‐00430p; IT‐003‐SW12p, Immune Technology, New York, NY, USA) were biotinylated with EZ‐link Sulfo‐NHS‐LC‐LC‐Biotin (21338, Thermo Fisher Scientific, Waltham, MA, USA). Removal of excess biotin was performed with Zeba Spin Desalting Columns (89883, Thermo Fisher Scientific). The biotinylated EBNA1 protein and pooled Influenza‐HA proteins were each coupled with fluorescent neutravidin‐labeled beads (antibody‐dependent cellular phagocytosis [ADCP]: F8776; antibody‐dependent complement deposition [ADCD]: F8775, Thermo Fisher Scientific).

### ADCP

ADCP was performed according to established protocols.^21^ In summary, 10μl of phosphate‐buffered saline (PBS) diluted patient plasma (1:50) was incubated with the bead‐coupled antigens for 2 hours at 37°C in V‐bottom plates. Unbound antibodies were removed by centrifugation. The 2 × 10^4^ THP‐1 cells (human monocytic leukemia cell line) dissolved in Roswell Park Memorial Institute (RPMI) media supplemented with 20% fetal calf serum (FCS) (R20) were added to each well to a final volume of 200μl and incubated for 4 hours at 37°C and 5% CO_2_. After incubation, cells were washed, and relative phagocytosis was measured by flow cytometry using the CytoFLEX (Beckman Coulter, Brea, CA, USA). Phagocytic scores were calculated by multiplying the percentage of bead‐positive THP‐1 cells by the median fluorescence intensity (MFI) of bead‐positive THP‐1 cells, then dividing by 10^6^. Each sample was analyzed in duplicate.

### ADCD

ADCD was performed as previously described.^22^ Briefly, patient plasma was diluted 1:10, and 10μl was incubated with 10μl of antigen‐coupled beads for 2 hours at 37°C. Following incubation, unbound antibodies were removed by washing. Lyophilized Low‐Tox guinea pig complement (CL4051, Cederlane, Burlington, ON, Canada) was reconstituted in 1ml of ice‐cold ddH_2_O and 200μl was added to each well. After a 15‐minute incubation at 37°C, the reaction was stopped by washing with PBS containing 15mM ethylenediaminetetraacetic acid (EDTA). Complement component 3 (C3) deposition on the beads was then stained with a 1:100 dilution of fluorescein isothiocyanate (FITC) conjugated anti‐guinea pig C3 polyclonal antibody (855385, MP Biomedicals, Santa Ana, CA, USA), and relative C3 deposition was analyzed by flow cytometry using the CytoFLEX instrument (Beckman Coulter, Brea, CA, USA). Complement deposition scores were calculated using the percentage of C3‐positive beads multiplied by the MFI of the C3‐positive beads and then divided by 10^4^. Each sample was analyzed in duplicate.

### Antibody‐Dependent Natural Killer Cell Activation

Antibody‐dependent natural killer cell activation (ADNKA) was performed as previously described.^23^ ELISA plates were coated with a protein‐PBS solution, including EBNA1 (4μg/ml) or pooled Influenza‐HA proteins (3μg/ml), and incubated overnight at 4°C. Subsequently, plates were blocked with 5% PBS‐bovine serum albumin (BSA). Plasma samples, diluted 1:60, were added to the plates and incubated for 2 hours at 37°C. Following this, 5 × 10^4^ natural killer (NK) cells per well were added, which had been previously isolated from buffy coats of 2 HCs using the NK Isolation Kit (130‐092‐657, Miltenyi, Bergisch‐Gladbach, Germany) and incubated overnight with 1ng/ml interleukin‐15 (IL‐15) at 37°C. The plates were incubated for 5 hours at 37°C in the presence of mouse anti‐CD107a (555802, BD Biosciences, Franklin Lakes, NJ, USA) as a label, along with Brefeldin A (420601, BioLegend, San Diego, CA, USA) and Golgi Stop (FisherScientific, 10716676) to accumulate intracellular cytokines. After incubation, the plates were washed and stained with surface markers anti‐CD56 (557747, BD Biosciences), anti‐CD16 (557758, BD Biosciences), and anti‐CD3 (557943, BD Biosciences) for 15 minutes at room temperature. The plates were washed and then incubated with FoxP3 fixation/permeabilization (Fix/Perm) solution (00‐5523‐00, Thermo Fisher Scient) at 4°C for 30 minutes to achieve permeabilization. For intracellular staining, mouse anti‐macrophage inflammatory protein‐1 β (MIP‐1β) (550078, BD Biosciences) and mouse anti‐interferon γ (IFN‐γ) (554702, BD Biosciences) were diluted 1:50 in Perm buffer and added to the plates for 15 minutes at room temperature. After a final wash, cells were resuspended in PBS and NK activation (MIP‐1β^+^, IFN‐γ^+^, CD107a^+^) was analyzed using flow cytometry with CytoFlex. The NK cells were identified by the CD3^−^CD16^+^CD56^+^ surface marker profile. Each plasma sample was analyzed in parallel using NK cells from both healthy donors, and values from both donors were averaged.

### Fc γ Receptor Binding

Fc γ receptor (FcγR) binding was analyzed using a custom multiplex Luminex assay, as previously described.^24^ In summary, EBNA1 (ab138345, abcam), EBV glycoprotein 350 (EBV‐gp350) (40373‐V08H, Sino Biological, Beijing, China) and a pool of 3 Influenza‐HA proteins (IT‐003‐B11p; IT‐003‐00430p; IT‐003‐SW12p, Immune Technology) were coupled to Luminex beads (MC10013‐01, MC10035‐01, MC10078‐01, Luminex, Austin, TX, USA). Diluted plasma samples (1:1000) were incubated with the prepared Luminex beads overnight at 4°C, with shaking, to allow immune complex formation. Phycoerythrin (PE)‐conjugated streptavidin was coupled to recombinant human FcγR (AVI‐tag biotinylation) and incubated with the samples for 2 hours. Binding of antigen‐specific antibodies to the respective FcγR was determined by the MFI signal on a ZE5 Cell Analyzer (Bio‐Rad, Hercules, CA, USA).

### MRI Assessment Methods

As described previously,^18^ annual brain MRI scans were conducted using a harmonized imaging protocol across all participating sites. The protocol included high‐resolution 3 dimensional (3D) magnetization prepared‐rapid gradient echo (MPRAGE), a 3D fluid attenuated inversion recovery (FLAIR) sequence, and a post‐contrast T1‐weighted sequence acquired at a spatial resolution of 1 mm^3^. T2 lesion volume (T2LV) was automatically quantified on FLAIR images using a multidimensional gated recurrent units‐based algorithm,^25^ with subsequent manual verification by experts. The number of CELs was determined through manual evaluation.

### Statistical Analysis

Demographic and clinical characteristics were summarized as counts and percentages for categorical variables and as medians with interquartile ranges (IQR) for continuous variables. Between‐group comparisons were performed using Fisher's exact test or the χ^2^ test for categorical variables (depending on sample size), and the Wilcoxon rank‐sum test for continuous variables, which were not normally distributed. Univariate comparisons of antibody features between groups were conducted using the Mann–Whitney *U* test for unpaired data (HC vs MS) and the Wilcoxon paired signed‐rank test for paired data (MS‐Active vs MS‐Remission). For comparisons across more than 2 groups, the Kruskal–Wallis test was applied followed by Dunn's multiple comparisons test. *p*‐values <0.05 were considered statistically significant. Boxplots were used to visualize distributional differences. These analyses were conducted in GraphPad Prism version 10.4.2 (GraphPad Software, Boston, MA). To assess associations between antibody features and continuous variables, Spearman correlation analyses were performed in R, and resulting *p*‐values were adjusted for multiple testing using the Benjamini–Hochberg procedure. Heatmaps of normalized antibody features were generated using the “*gplots*” package. Values were z‐scored, missing values were imputed using the R package “*missForest*” and clustering was disabled. To visualize relative feature enrichment between groups, percentile ranks were calculated across all individuals to allow direct comparison across features. In the resulting flower plots, petal length represents the mean percentile rank of each feature, with longer petals indicating higher relative values within the group. Missing values were imputed using the R package “*missForest*”. To assess group differences in antibody features, log‐transformed signal intensities served as dependent variables in linear regression or linear mixed‐effects models, depending on the comparison. For MS versus HC, linear regression adjusted for age and sex was used. For paired MS‐Active versus MS‐Remission comparisons, mixed models were run including age, sex, body mass index, disease duration, EDSS, and treatment as fixed effects, with patient identification as a random intercept. All estimates were back‐transformed and represent percent changes in the geometric mean of the antibody feature levels per unit change in the independent variable. Models were implemented using the *lme4* package in R version 4.3.3. All other statistical analyses and data preprocessing were performed using R version 4.4.2 (R Core Team, Vienna, Austria).

## Results

### Pro‐Inflammatory Fc Signature of EBNA1‐Specific Antibodies in MS

**FIGURE 1:**
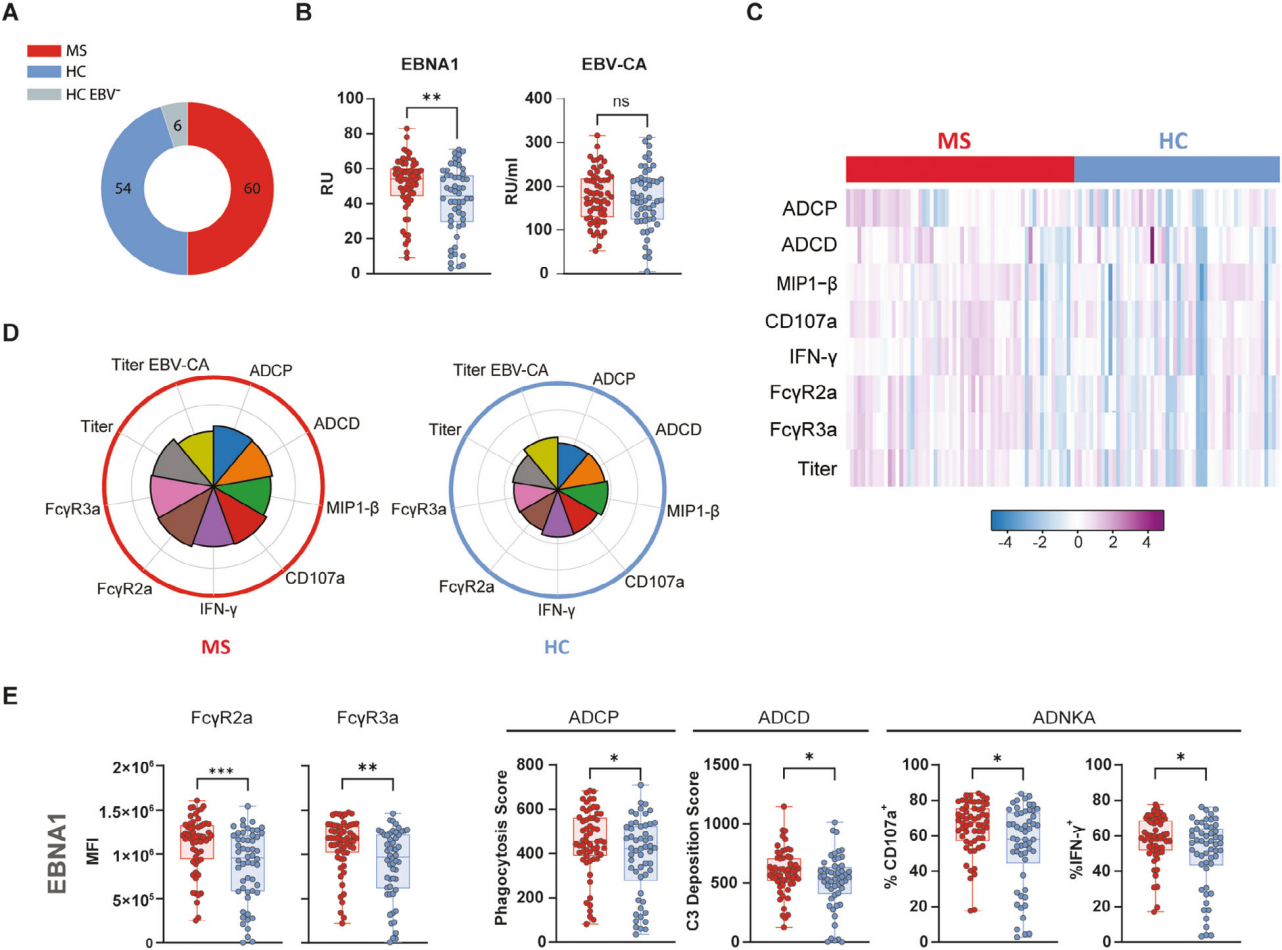
Pro‐inflammatory Fc signature of Epstein–Barr virus (EBV) nuclear antigen 1 (EBNA1)‐specific antibodies in multiple sclerosis (MS) (A) EBV seropositivity rates in MS patients during active disease and in healthy controls (HCs). All MS patients, 100% (60/60) were EBV‐seropositive. Among HCs, 90% (54/60) were EBV‐seropositive, and 10% (6/60) were EBV‐seronegative. Patients were considered seropositive if they tested positive for at least EBNA1 or EBV‐capsid antigen (EBV‐CA) antibodies. (B) Boxplots showing the distribution of EBNA1 immunoglobulin G (IgG) (RU) and EBV‐CA IgG (RU/ml) levels in MS patients and EBV seropositive HCs. Each dot represents one patient. Differences were compared by the Mann–Whitney *U* test, *p* values indicate statistical significance (*p ≤ 0.05, **p ≤ 0.01, and ***p ≤ 0.001). (C) Heatmap of z‐scored values for EBNA1‐specific antibody‐mediated effector functions (antibody‐dependent complement deposition [ADCD], antibody‐dependent cellular phagocytosis [ADCP], and antibody‐dependent natural killer cell activation [ADNKA]), Fc γ receptor (FcγR) binding capacity (FcγR2A, FcγR3A) and EBNA1 titer (Titer) in MS patients during active disease and EBV seropositive HCs. (D) Flower plots visualizing EBNA1‐specific antibody‐mediated effector functions (ADCD, ADCP, and ADNKA), FcγR binding capacity (FcγR2A, FcγR3A) and EBNA1 titer (Titer) in MS patients during active disease compared to EBV seropositive HCs. Petal length represents the mean percentile rank of each feature, with longer petals indicating higher relative values within the group. Percentile ranks were calculated across all individuals to allow direct comparison across features. (E) Univariate analysis of EBNA1 antibody FcγR binding capacity (FcγR2A, FcγR3A), as well as effector functions (ADCD, ADCP, and ADNKA). Boxplots display the data distribution, showing the median (line within the box), interquartile range (IQR) (box), and minimum to maximum values (whiskers); each data point represents an individual patient. The Mann–Whitney *U* test was used for statistical comparisons between groups (see also Table S1), *p* values indicate statistical significance (*p ≤ 0.05, **p ≤ 0.01, and ***p ≤ 0.001).

We enrolled 60 individuals with relapse‐onset MS. Blood samples were collected during active disease phases, defined as either clinical relapse or the presence of contrast‐enhancing lesions on MRI. A control group of 60 healthy individuals, matched by sex and age, was also included (Table 1). EBV‐specific antibodies were detectable in all MS patients (100%) and in 54 of 60 (90%) HCs (Fig 1A). For the following experiments, only samples from seropositive individuals were analyzed. Accordingly, 6 of the 60 HCs were excluded from further analyses. EBNA1‐specific antibody titers were significantly higher in MS patients compared to healthy virus carriers (median [IQR]: 54.0 [44.5–60.0] relative units (RU) vs 44.5 [29.5–56.25] RU; fold change = 1.21; *p* = 0.01). In contrast, titers against the EBV‐CA did not differ significantly between the groups (median [IQR]: 174.0 [130.0–217.5] RU/ml vs 167.5 [123.6–215.4] RU/ml; *p* = 0.78) (Table S1) (see Fig 1B).

We next investigated whether biophysical and functional features of EBNA1‐specific antibodies differ between individuals with MS and healthy EBV carriers. To this end, we comprehensively assessed EBNA1‐IgG effector functions, including ADCP, ADCD, and ADNKA. We also evaluated the interaction of antigen‐specific antibodies with activating FcγRs, including FcγR2A and FcγR3A (see Fig 1C, D).

EBNA1‐specific antibodies from MS patients exhibited increased binding to all tested FcγRs compared to those from HCs (see Fig 1E). This enhanced receptor binding was consistently associated with greater EBNA1 IgG‐Fc effector functions—namely ADCD, ADCP, and ADNKA (see Fig 1E). Linear regression analyses adjusted for age and sex confirmed consistent increases across all functional assays and FcγR affinity parameters. Functional responses were elevated by up to 44% (ADCD), and FcγR binding increased by as much as 60% (FcγR2A) in MS patients relative to HCs (Table 2). We further performed linear regression analyses that accounted for age, sex, and EBNA1 titers to investigate whether the observed functional differences were associated with higher concentrations of EBNA1‐specific antibodies in MS patients. After adjusting for antibody concentration, the differences in Fc‐mediated effector functions between MS patients and HCs decreased and were no longer statistically significant (Table S2).

**TABLE 2:** Linear Regression Analysis to Compare Antibody Profiles of MS Patients to Healthy Controls

Variable	Estimate CI (95%)	*p*
EBNA1		
Titer	1.36 [1.08–1.72]	**0.010**
ADCP	1.30 [1.04–1.62]	**0.022**
ADCD	1.44 [1.04–2.01]	**0.030**
ADNKA CD107a	1.33 [1.06–1.67]	**0.014**
ADNKA IFN‐γ	1.29 [1.04–1.61]	**0.021**
FcγR2a	1.60 [1.17–2.19]	**0.004**
FcγR3a	1.48 [1.13–1.94]	**0.005**
Influenza‐HA		
ADCP	0.84 [0.69–1.02]	**0.082**
ADCD	0.69 [0.41–1.17]	0.168
ADNKA CD107a	1.34 [0.93–1.94]	0.112
ADNKA IFN‐γ	1.20 [0.84–1.72]	0.305
FcγR2a	0.79 [0.60–1.05]	0.104
FcγR3a	0.76 [0.53–1.09]	0.133
EBV‐gp350		
FcγR2a	1.23 [0.80–1.89]	0.335
FcγR3a	1.25 [0.81–1.93]	0.309
EBV‐CA		
Titer	1.13 [0.92–1.38]	0.229

HC serves as a reference group. Back‐transformed estimates reflect percent change relative to HC (estimate of 1.20 = 20% increase). Models adjusted for age and sex. Bold values indicate statistically significant *p*‐values (*p* < 0.05).

ADCD = antibody‐dependent complement deposition; ADCP = antibody‐dependent cellular phagocytosis; ADNKA = antibody‐dependent natural killer cell activation; EBNA1 = Epstein–Barr virus nuclear antigen 1; EBV‐CA = Epstein–Barr virus capsid antigen; FcγR = Fc γ receptor; gp350 = glycoprotein 350; HC = healthy control; IFN‐γ = interferon‐γ; Influenza‐HA = influenza hemagglutinin.

**FIGURE 2:**
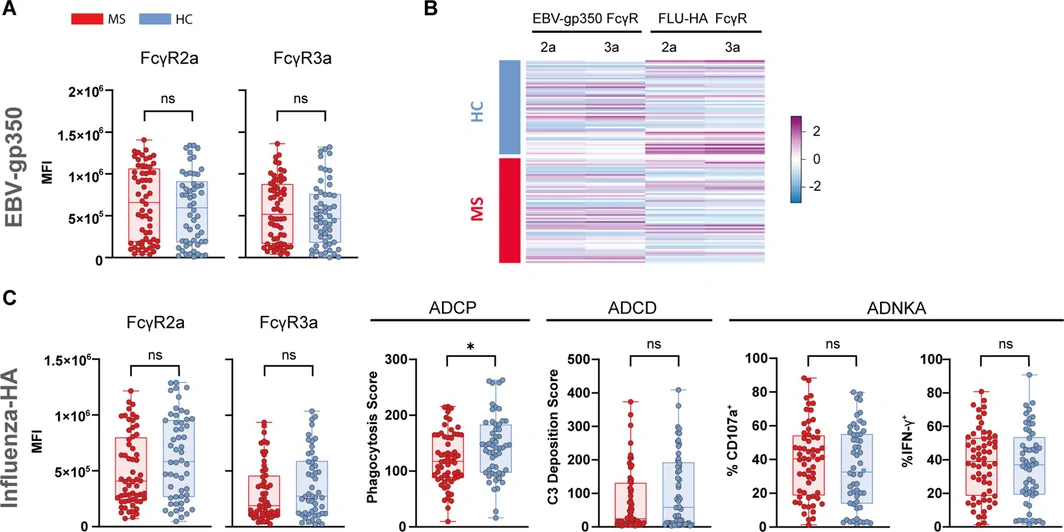
Fc γ receptor (FcγR) binding to antibodies against viral antigens other than Epstein–Barr virus (EBV) nuclear antigen 1 (EBNA1). (A) Univariate analysis of EBV‐glycoprotein 350 (EBV‐gp350) antibody binding capacity to FcγRs. Boxplots display the data distribution, showing the median (line within the box), interquartile range (IQR) (box), and minimum to maximum values (whiskers); each data point represents an individual patient. The Mann–Whitney *U* test was used to compare groups. No statistically significant differences were observed (ns). (B) Heatmap of z‐scored values representing EBV‐gp350 and influenza hemagglutinin (Influenza‐HA) antibody binding capacity to FcγRs in MS patients during active disease (n = 60) and EBV seropositive healthy controls (HCs) (n = 54). (C) Univariate analysis of Influenza‐HA antibody binding capacity to FcγRs. Boxplots show the data distribution, with each point representing an individual patient. Differences were compared by the Mann–Whitney‐*U* test; *p* values indicate statistical significance (*p ≤ 0.05) (see also Table S1).

To determine whether the elevated EBNA1‐specific humoral responses in MS were antigen‐specific, we also analyzed IgG responses to EBV‐gp350 (expressed during lytic replication of EBV) and Influenza‐HA. Neither EBV‐gp350‐ nor Influenza‐HA‐specific antibodies showed significant differences in FcγR binding between MS patients and HCs, based on both univariate and regression analyses (Fig 2A–C, Table 2). Similarly, antibody‐mediated effector functions (ADCD, ADNKA) against Influenza‐HA were comparable between groups. A modest increase in ADCP was observed in HCs compared to MS patients, but this difference lost significance after adjustment for age and sex (see Fig 2C, Table 2). These findings indicate that the enhanced Fc‐mediated IgG responses and FcγR binding observed in MS are specific to EBNA1.

### Increased FcγR Binding of EBV‐Specific IgG Correlates with Disease Activity in MS

To further explore whether enhanced EBNA1‐specific antibody responses in MS patients correlate with disease activity, we simultaneously analyzed paired serum samples from the 60 patients included in our study (120 total samples), collected during both active disease (MS‐Active, defined as clinical relapse and/or contrast‐enhancing brain lesions) and remission (MS‐Remission).

**FIGURE 3:**
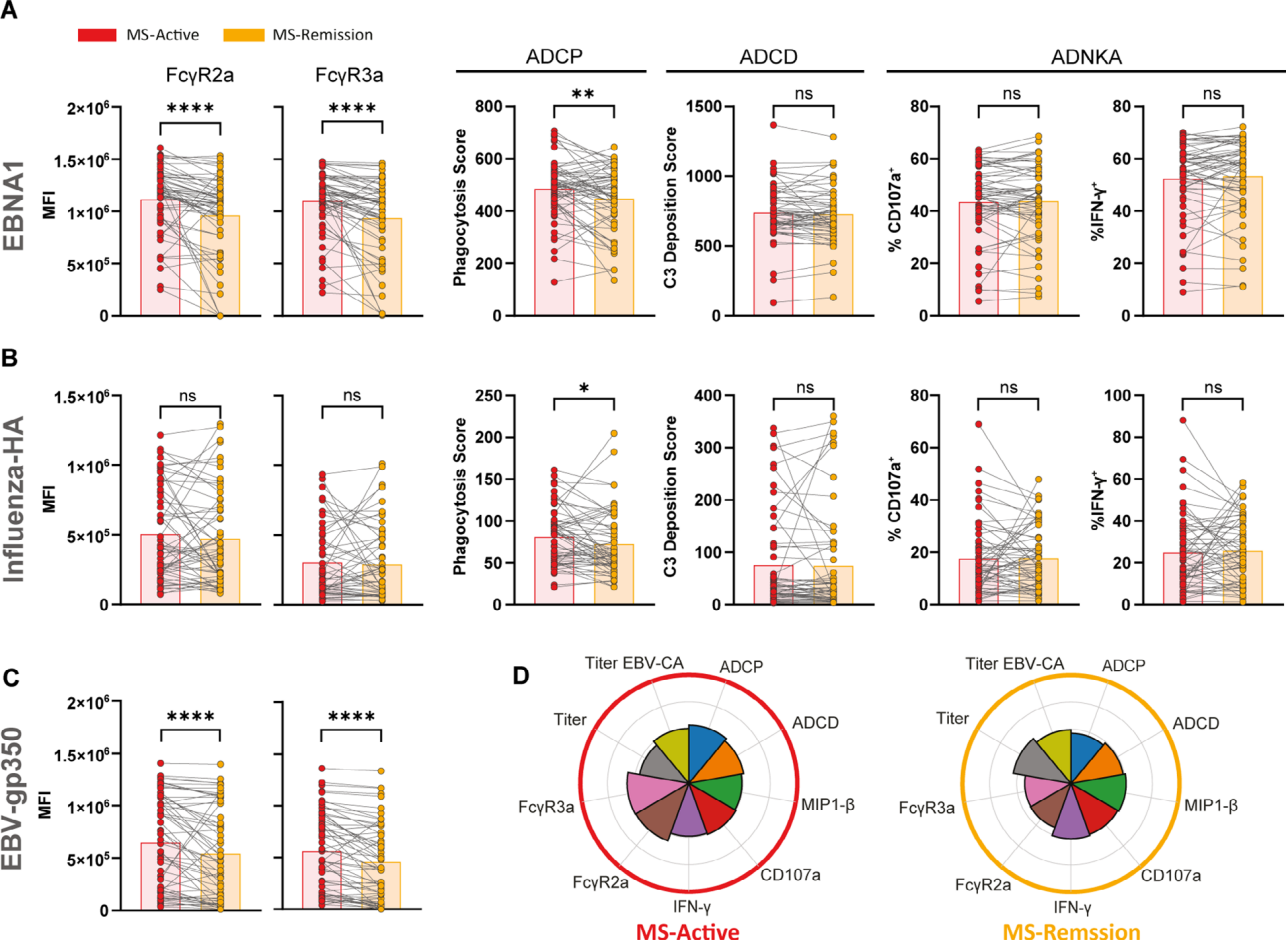
Increased Epstein–Barr virus (EBV)‐immunoglobulin G (IgG) binding to Fc γ receptors (FcγR) is associated with focal disease activity in multiple sclerosis (MS). (A–C) Univariate analysis of EBV nuclear antigen 1 (EBNA1), influenza hemagglutinin (Influenza‐HA), and EBV‐glycoprotein 350 (EBV‐gp350) antibody binding capacity to FcγR, as well as their effector functions (antibody‐dependent complement deposition [ADCD], antibody‐dependent cellular phagocytosis [ADCP], and antibody‐dependent natural killer cell activation [ADNKA]). Bar plots display the mean of each group; each data point represents an individual patient, and paired values are connected by lines. Paired samples were collected from the same MS patients during active disease (MS‐Active, n = 60; red) and remission (MS‐Remission, n = 60; yellow). The Mann–Whitney *U* test was used to compare groups (see Table S1). *p*‐Values indicate statistical significance (*p ≤ 0.05, **p ≤ 0.01, ***p ≤ 0.001, and ****p ≤ 0.0001). (D) Flower plots visualizing EBNA1‐specific antibody‐mediated effector functions (ADCD, ADCP, and ADNKA), FcγR binding capacity and EBNA1 titer (Titer) in MS‐Active and MS‐Remission. Petal length represents the mean percentile rank of each feature, with longer petals indicating higher relative values within the group. Percentile ranks were calculated across all individuals to allow direct comparison across features.

EBNA1 antibody titers did not significantly differ between these states (median [IQR]: active 60 [44.5–60] RU vs remission 60 [44–64.75] RU; *p* = 0.17). In contrast, EBNA1‐IgG binding affinity to all tested activating FcγRs was higher during active disease. This increase ranged from 55% for FcγR3A to 88% for FcγR2A in patients exhibiting focal disease activity (Fig 3A, D, Table 3). Among Fc‐mediated functions, ADCP showed an increase during active disease, while ADCD and ADNKA remained largely unchanged. Notably, this elevated FcγR binding was not limited to EBNA1‐specific antibodies, and antibodies targeting EBV‐gp350 demonstrated increased FcγR affinity during focal disease. By contrast, antibody responses to Influenza‐HA remained stable regardless of disease activity (see Fig 3B, C). As some patients received a different treatment during remission compared with active disease, we performed an additional analysis restricted to patients who remained within the same treatment category at both time points. In this subset, the increased FcγR binding of EBNA1‐specific and EBV‐gp350‐specific antibodies during active disease remained statistically significant (Table S3, Fig S1). To address the potential effects of heterogeneity in disease activity and its temporal proximity to the time of sample collection, we ran additional stratified analyses and correlation analyses. Neither the type of disease activity (clinical relapse and/or CELs) nor the time interval between sample collection and the last episode of disease activity was associated with differences in antibody profiles (Tables S4 and S5).

**TABLE 3:** Group Comparison of Antibody Profiles Between MS‐Active and MS‐Remission using a Multivariable Mixed Linear Model

Variable	Estimate CI (95%)	*p*
EBNA1		
Titer	0.99 [0.91–1.07]	0.791
ADCP	1.08 [1.00–1.16]	**0.044**
ADCD	0.99 [0.95–1.03]	0.647
ADNKA CD107a	0.99 [0.92–1.05]	0.682
ADNKA IFN‐γ	0.98 [0.93–1.03]	0.415
FcγR2a	1.88 [1.19–2.93]	**0.008**
FcγR3a	1.55 [1.17–2.06]	**0.003**
Influenza‐HA		
ADCP	1.14 [1.02–1.28]	**0.026**
ADCD	1.05 [0.87–1.27]	0.615
ADNKA CD107a	1.01 [0.83–1.24]	0.914
ADNKA IFN‐γ	0.98 [0.80–1.21]	0.880
FcγR2a	1.16 [0.98–1.37]	0.087
FcγR3a	1.10 [0.90–1.36]	0.365
EBV‐gp350		
FcγR2a	1.56 [1.22–1.98]	**<0.001**
FcγR3a	1.97 [1.33–2.86]	**0.001**
EBV‐CA		
Titer	1.03 [0.98–1.08]	0.243

MS‐Remission serves as the reference group. Back‐transformed estimates show percent change relative to remission (estimate of 1.20 = 20% increase). Models adjusted for sex, age, BMI, disease duration, EDSS, and treatment. Bold values indicate statistically significant *p*‐values (*p* < 0.05).

ADCD = antibody‐dependent complement deposition; ADCP = antibody‐dependent cellular phagocytosis; ADNKA = antibody‐dependent natural killer cell activation; BMI = body mass index; CI = confidence interval; EBNA1 = Epstein–Barr virus nuclear antigen 1; EBV‐CA = Epstein–Barr virus capsid antigen; EDSS = Expanded Disability Status Scale; FcγR = Fc γ receptor; gp350 = glycoprotein 350; IFN‐γ = interferon‐γ; Influenza‐HA = influenza hemagglutinin; MS = multiple sclerosis.

## Discussion

Our study demonstrates that EBNA1‐specific antibodies in people with MS exhibit enhanced IgG Fc‐mediated proinflammatory activity compared to those in healthy EBV carriers. Moreover, we identify an EBV‐specific serological signature—characterized by increased binding of EBV‐specific IgG to FcγRs—that correlates with focal disease activity.

Previous studies have primarily focused on the seroprevalence and titers of EBV‐ and EBNA1‐specific antibodies as markers of susceptibility to MS. In contrast, our data extend these observations by identifying qualitative differences in FcγR binding and Fc‐mediated effector functions that are associated with clinical activity after disease onset. These findings suggest that not only the quantity, but also the functional profile of EBV‐specific IgG is relevant to MS biology and further support the link between EBV‐directed adaptive immunity and disease activity.

The increased effector responses and FcγR binding of EBV‐specific antibodies observed in patients with MS may, in part, be driven by higher antibody concentrations, as normalization to EBNA1‐specific IgG levels attenuated these differences. However, antibody activity reflects the combined effects of multiple features, including concentration, antigen specificity and affinity, Fc receptor binding affinity, and effector functions. Our functional assays are designed to capture this integrated activity.

A recent study examining the functional characteristics of EBNA1‐specific IgG in young adults during primary EBV infection, infectious mononucleosis (IM),^16^ a condition associated with a 2‐ to 3‐fold increased risk of developing MS, supports our findings.^26^ In this study, EBNA1‐specific IgG capable of binding activating FcγRs were detected at 6 months and 1 year after onset of IM. As noted by the authors, this contrasts with findings from certain vaccine studies,^27^ where antigen‐binding IgG were reported to remain stable, but FcγR‐binding antibodies declined over time. EBNA1‐specific IgG was also capable of eliciting Fc‐mediated effector functions such as ADCP and ADCD,^16^ which demonstrates that long‐lasting Fc‐mediated effector functions of EBNA1‐specific antibodies are established during IM.^16^

EBNA1‐specific antibodies are considered pathogenetically relevant in MS because of their ability to cross‐react with antigens expressed in the CNS such as anoctamin‐2, α‐B crystallin (αB‐crystallin), and glial cellular adhesion molecule.^12^, ^13^, ^14^, ^15^ Elevated antibody titers against the aforementioned antigens have been associated with an increased risk of developing MS, with the strongest risk observed in individuals who are human leukocyte antigen (HLA)‐DRB1*15:01 positive.^15^ The impact of HLA class II in this context suggests that EBV‐specific CD4^+^ T cells may also contribute to CNS antigen misrecognition, implying that molecular mimicry extends beyond antibody responses.

EBNA1 is indeed the most consistently recognized CD4^+^ T cell‐antigen encoded by EBV.^28^ EBNA1‐specific CD4^+^ T cells, increased in frequency in MS patients,^29^ cross‐recognize CNS myelin antigens,^11^ and cross‐reactivity has also been observed for T cells targeting lytic EBV antigens.^30^

FcγRs facilitate internalization of opsonized material or immune complexes and have a crucial role in augmenting antigen presentation to both CD4^+^ and CD8^+^ T cells.^31^, ^32^, ^33^, ^34^, ^35^, ^36^, ^37^, ^38^, ^39^, ^40^, ^41^ Building on this, enhanced FcγR binding may promote immune complex uptake and antigen presentation, thereby facilitating CD4^+^ T‐cell activation. Likewise, EBNA1‐specific antibodies that cross‐recognize aforementioned encephalitogenic antigens may facilitate the targeting of molecular mimicry epitopes to major histocompatibility complex class II loading compartments, an effect potentially amplified by enhanced Fc receptor binding, and thereby promote cross‐reactive CD4^+^ T‐cell responses.

Although the MS disease trait is predominantly linked to deregulated IgG responses to EBNA1,^3^, ^4^, ^5^, ^6^, ^7^, ^8^, ^9^, ^10^ we observed that focal disease activity as defined by presence of clinical relapses or Gd+ MRI lesions is additionally associated with higher FcγR binding capacity of antibodies against EBV‐gp350, expressed during lytic viral replication. Dysregulation of intrinsic B cell control of EBV gene expression has recently been shown to augment lytic replication of EBV in people with MS.^42^, ^43^ In spontaneous lymphoblastoid cell lines isolated from MS patients, higher EBV lytic gene expression was reported for patients during active compared to stable disease^42^ and CD8^+^ T cells targeting lytic EBV antigens were found to be increased in frequency in patients with active compared to stable disease.^44^ Our findings corroborate these observations and support the notion that focal MS disease activity is associated with aberrant immune responses to lytic EBV antigens, in addition to the MS disease‐trait associated increase in immune responses to EBNA1. However, a direct causal role for these antibody functions in disease pathogenesis remains to be established.

Our study has several limitations. Because of the relatively small sample size, the findings from this exploratory analysis require validation in larger patient cohorts. Ideally, such cohorts should be recruited before the initiation of immunotherapy and followed longitudinally over an extended period, incorporating not only markers of focal disease activity, but also measures that capture progression independent of relapse activity. Additionally, although our study suggests a potential role for EBNA1‐specific T‐cell responses, these were not directly investigated. Enhanced EBNA1‐specific antibody responses in MS may instead represent a surrogate marker of increased CD4^+^ T‐cell responses, which may, at least in part, be driven by cross‐recognition of MS‐relevant antigens.^11^, ^45^ Future systems immunology studies integrating both cellular and humoral immune features in response to EBV infection may provide critical insights into the relationship between EBV and MS disease trajectories and support the development of more targeted therapeutic strategies.

Taken together, our study indicates that the association between elevated EBNA1‐specific antibody responses and MS susceptibility is driven by a combination of both fragment antigen–binding‐mediated and Fc‐mediated mechanisms with Fc effector functions potentially contributing to focal disease activity. Future studies should ideally incorporate analyses of both humoral and cellular immune responses to EBV infection, which could yield granular insights into the relationship between EBV and MS disease trajectories and identification of novel targets to restrain EBV‐induced hyperactive immune responses in MS.

## Author Contributions

S.S., C.M., G.L., Y.B., J.K., and J.D.L. contributed to the conception and design of the study; M.B., P.T.N., E.A.J.W., S.S., M.C., G.L., Y.B., J.K., and J.D.L. contributed to the acquisition and analysis of data; M.B., P.T.N., E.A.J.W., S.S., J.K., and J.D.L. contributed to drafting the text or preparing the figures.

## Potential Conflicts of Interest

Nothing to report.

## Supporting information


**Figure S1.** Increased EBV‐IgG binding to Fcγ receptors is associated with focal disease activity in MS under stable treatment. Univariate analysis of EBNA1, Influenza‐HA, and gp350 antibody binding capacity to FcγR, as well as their effector functions (ADCD, ADCP, and ADNKA). Bar plots display the mean of each group; each data point represents an individual patient, and paired values are connected by lines. Paired samples were collected from the same MS patients during active disease (MS‐Active, *n* = 60; red) and remission (MS‐Remission, *n* = 60; yellow). At both time points, patients remained within the same treatment category. The Mann–Whitney *U* test was used to compare groups (see also Table S1). *p*‐Values indicate statistical significance (*p ≤ 0.05, **p ≤ 0.01, ***p ≤ 0.001 and ****p ≤ 0.0001).
**Table S1.** Exact *p*‐values from Mann–Whitney *U* and Wilcoxon paired signed‐rank tests.
**Table S2.** Linear regression analysis to compare antibody profiles of MS patients to healthy controls with titer adjustment.
**Table S3.** Group comparison of antibody profiles between MS‐Active and MS‐Remission patients under stable treatment using a multivariable mixed linear model.
**Table S4.** Spearman correlations between antibody profiles and Δ time since the most recent relapse or MRI‐detected new gadolinium‐enhancing lesions.
**Table S5.** Kruskal–Wallis tests comparing antibody profiles across patients grouped by disease activity type.

## Data Availability

Processed datasets supporting the results of this study are available from the corresponding author on request.
