# Supplementary material for: Proinflammatory Epstein–Barr Virus Antibody Functions Track with Disease Activity in Multiple Sclerosis
Source: Ann Neurol. 2026 Jun 17;100(3):547–58. doi: 10.1002/ana.78285 (PMC13495917; doi:10.1002/ana.78285)
Supplement: Supplementary file 1 [file ANA-100-547-s001.docx]

**Supplementary Table 1:** **Exact p-values from Mann–Whitney U and Wilcoxon paired signed-rank tests.**

| HC vs. MS (Mann-Whitney U test) | | | | |
| --- | --- | --- | --- | --- |
|  |  | **EBNA1** | **EBV-gp350** | **Influenza-HA** |
|  | Titer | 0.01 |  |  |
|  | ADCP | 0.037 |  | 0.031 |
|  | ADCD | 0.044 |  | 0.290 |
|  | ADNKA CD107a | 0.013 |  | 0.368 |
|  | ADNKA IFN-γ | 0.032 |  | 0.552 |
|  | FcγR2a | 0.001 | 0.351 | 0.133 |
|  | FcγR3a | 0.002 | 0.511 | 0.225 |
| MS-Active vs. MS Remission (Wilcoxon paired signed-rank test) | | | | |
|  | Titer | 0.028 |  |  |
|  | ADCP | 0.008 | 0.010 |  |
|  | ADCD | 0.267 | 0.436 |  |
|  | ADNKA CD107a | 0.836 | 0.700 |  |
|  | ADNKA IFN-γ | 0.470 | 0.802 |  |
|  | FcγR2a | <0.001 | 0.236 | <0.001 |
|  | FcγR3a | <0.001 | 0.854 | <0.001 |

Abbreviations: ADCP, antibody-dependent cellular phagocytosis; ADCD, antibody-dependent complement deposition; ADNKA, antibody-dependent natural killer cell activation; IFN- γ, interferon-gamma; FcγR, Fc gamma receptor; EBNA1, Epstein-Barr virus nuclear antigen 1; Influenza-HA, Influenza hemagglutinin; EBV-gp350, Epstein–Barr virus glycoprotein 350; EBV-CA, Epstein–Barr virus capsid antigen

**Supplementary Table 2: Linear regression analysis to compare antibody profiles of MS patients to healthy controls with titer adjustment.**

| Variable | | Estimate | CI (95%) | p value |
| --- | --- | --- | --- | --- |
| EBNA1 | | | | |
|  | ADCP | 1.13 | [0.92;1.39] | 0.228 |
|  | ADCD | 1.03 | [0.83;1.29] | 0.779 |
|  | ADNKA CD107a | 1.09 | [0.92;1.31] | 0.320 |
|  | ADNKA IFN-γ | 1.08 | [0.91;1.29] | 0.390 |
|  | FcγR2a | 1.18 | [0.95;1.47] | 0.140 |
|  | FcγR3a | 1.11 | [0.94;1.32] | 0.216 |

HC serves as a reference group. Back-transformed estimates reflect percent change relative to HC (estimate of 1.20 = 20% increase). Models adjusted for Titer, age and sex.
Abbreviations: ADCP, antibody-dependent cellular phagocytosis; ADCD, antibody-dependent complement deposition; ADNKA, antibody-dependent natural killer cell activation; IFN- γ, interferon-gamma; FcγR, Fc gamma receptor; EBNA1, Epstein-Barr virus nuclear antigen 1; Influenza-HA, Influenza hemagglutinin;

**Supplementary Table 3:** **Group comparison of antibody profiles between MS-Active and MS-Remission patients under stable treatment using a multivariable mixed linear model.**

| Variable | | Estimate | CI (95%) | p value |
| --- | --- | --- | --- | --- |
| EBNA1 | | | | |
|  | Titer | 0.99 | [0.89;1.10] | 0.780 |
|  | ADCP | 1.10 | [0.99;1.22] | 0.072 |
|  | ADCD | 0.98 | [0.94;1.02] | 0.378 |
|  | ADNKA CD107a | 0.98 | [0.89;1.09] | 0.708 |
|  | ADNKA IFN-γ | 0.97 | [0.90;1.03] | 0.318 |
|  | FcγR2a | 2.39 | [1.13;5.04] | **0.023** |
|  | FcγR3a | 1.75 | [1.10;2.78] | **0.021** |
| Influenza-HA | | | | |
|  | ADCP | 1.14 | [1.02;1.28] | **0.026** |
|  | ADCD | 1.16 | [0.96;1.39] | 0.124 |
|  | ADNKA CD107a | 0.98 | [0.75;1.27] | 0.845 |
|  | ADNKA IFN-γ | 0.92 | [0.71;1.21] | 0.555 |
|  | FcγR2a | 1.20 | [0.98;1.44] | 0.080 |
|  | FcγR3a | 1.17 | [0.93;1.47] | 0.184 |
| EBV-gp350 | | | | |
|  | FcγR2a | 1.65 | [1.67;2.33] | **0.006** |
|  | FcγR3a | 2.37 | [1.31;4.28] | **0.006** |
| EBV-CA | | | | |
|  | Titer | 1.02 | [0.96;1.08] | 0.532 |

MS-Remission serves as the reference group. Back-transformed estimates show percent change relative to remission (estimate of 1.20 = 20% increase). Models adjusted for sex, age, BMI, disease duration, EDSS, and treatment.

Abbreviations: ADCP, antibody-dependent cellular phagocytosis; ADCD, antibody-dependent complement deposition; ADNKA, antibody-dependent natural killer cell activation; IFN- γ, interferon-gamma; FcγR, Fc gamma receptor; EBNA1, Epstein-Barr virus nuclear antigen 1; Influenza-HA, Influenza hemagglutinin; EBV-gp350, Epstein–Barr virus glycoprotein 350; EBV-CA, Epstein–Barr virus capsid antigen; CI, confidence interval

**EBV-gp350**

**Supplementary Figure 1 Increased EBV-IgG binding to Fcγ receptors is associated with focal disease activity in MS under stable treatment.** Univariate analysis of EBNA1, Influenza-HA, and gp350 antibody binding capacity to FcγR, as well as their effector functions (ADCD, ADCP, and ADNKA). Bar plots display the mean of each group; each data point represents an individual patient, and paired values are connected by lines. Paired samples were collected from the same MS patients during active disease (MS-Active, n=60; red) and remission (MS-Remission, n=60; yellow). At both time points, patients remained within the same treatment category. The Mann-Whitney U test was used to compare groups (see also Table S1). P-values indicate statistical significance (*p ≤ 0.05, **p ≤ 0.01, ***p ≤ 0.001 and ****p≤ 0.0001).

**Supplementary Table 4: Spearman correlations between antibody profiles and Δ time since the most recent relapse or MRI-detected new gadolinium-enhancing lesions.**

| Variable | | Spearman ρ | p-value | FDR corrected p-value |
| --- | --- | --- | --- | --- |
| EBNA1 | | | | |
|  | Titer | 0.193 | 0.139 | 0.356 |
|  | ADCP | 0.121 | 0.359 | 0.506 |
|  | ADCD | 0.123 | 0.351 | 0.506 |
|  | ADNKA CD107a | 0.193 | 0.139 | 0.356 |
|  | ADNKA IFN-γ | 0.279 | 0.031 | 0.132 |
|  | FcγR2a | 0.306 | 0.018 | 0.132 |
|  | FcγR3a | 0.285 | 0.027 | 0.132 |
| Influenza-HA | | | | |
|  | ADCP | 0.187 | 0.153 | 0.356 |
|  | ADCD | 0.105 | 0.426 | 0.568 |
|  | ADNKA CD107a | 0.145 | 0.270 | 0.432 |
|  | ADNKA IFN-γ | 0.182 | 0.163 | 0.356 |
|  | FcγR2a | 0.172 | 0.188 | 0.376 |
|  | FcγR3a | 0.089 | 0.516 | 0.587 |
| EBV-gp350 | | | | |
|  | FcγR2a | 0.091 | 0.488 | 0.587 |
|  | FcγR3a | 0.071 | 0.589 | 0.589 |
| EBV-CA | | | | |
|  | Titer | 0.076 | 0.563 | 0.587 |

Shown are Spearman ρ, unadjusted p-values, and FDR-corrected p-values (Benjamini–Hochberg method). Abbreviations: ADCP, antibody-dependent cellular phagocytosis; ADCD, antibody-dependent complement deposition; ADNKA, antibody-dependent natural killer cell activation; IFN- γ, interferon-gamma; FcγR, Fc gamma receptor; EBNA1, Epstein-Barr virus nuclear antigen 1; Influenza-HA, Influenza hemagglutinin; EBV-gp350, Epstein–Barr virus glycoprotein 350; EBV-CA, Epstein–Barr virus capsid antigen; BH, Benjamini–Hochber

**Supplementary Table 5: Kruskal–Wallis tests comparing antibody profiles across patients grouped by disease activity type.**

| Variable | | Kruskal-Wallis p |  |
| --- | --- | --- | --- |
| EBNA1 | | | |
|  | Titer | 0.3753 |  |
|  | ADCP | 0.7954 |  |
|  | ADCD | 0.8324 |  |
|  | ADNKA CD107a | 0.2824 |  |
|  | ADNKA IFN-γ | 0.1684 |  |
|  | FcγR2a | 0.3895 |  |
|  | FcγR3a | 0.487 |  |
| Influenza-HA | | | |
|  | ADCP | 0.5989 |  |
|  | ADCD | 0.295 |  |
|  | ADNKA CD107a | 0.4525 |  |
|  | ADNKA IFN-γ | 0.3102 |  |
|  | FcγR2a | 0.928 |  |
|  | FcγR3a | 0.9331 |  |
| EBV-gp350 | | | |
|  | FcγR2a | 0.4892 |  |
|  | FcγR3a | 0.3171 |  |
| EBV-CA | | | |
|  | Titer | 0.0449 |  |

Disease activity type includes clinical relapse only, new gadolinium-enhancing lesions on MRI only, or both a clinical relapse and new MRI lesions. Shown are the antibody profiles and the corresponding Kruskal–Wallis p-values.
Abbreviations: ADCP, antibody-dependent cellular phagocytosis; ADCD, antibody-dependent complement deposition; ADNKA, antibody-dependent natural killer cell activation; IFN- γ, interferon-gamma; FcγR, Fc gamma receptor; EBNA1, Epstein-Barr virus nuclear antigen 1; Influenza-HA, Influenza hemagglutinin; EBV-gp350, Epstein–Barr virus glycoprotein 350; EBV-CA, Epstein–Barr virus capsid antigen
